# Validation of Non-empirical Fat-free Mass Estimation Model for a Wrist-worn Device

**DOI:** 10.2478/joeb-2022-0006

**Published:** 2022-06-25

**Authors:** Aleksandr Polokhin, Anna Pronina, Andrey Boev, Stas Gorbunov

**Affiliations:** 1 AURA Devices, Inc., Wilmington, DE, USA

**Keywords:** Bioelectrical impedance analysis, fat free mass, upper-body measurement, multiple linear regression, predictive modeling

## Abstract

Fat-free mass (FFM) estimation has dramatic importance for body composition evaluation, often providing a basis for treatment of obesity and muscular dystrophy. However, current methods of FFM estimation have several drawbacks, usually related to either cost-effectiveness and equipment size (dual-energy X-ray absorptiometry (DEXA) scan) or model limitations. In this study, we present and validate a new FFM estimation model based on hand-to-hand bioimpedance analysis (BIA) and arm volume. Forty-two participants underwent a full-body DEXA scan, a series of anthropometric measurements, and upper-body BIA measurements with the custom-designed wearable wrist-worn impedance meter. A new two truncated cones (TTC) model was trained on DEXA data and achieved the best performance metrics of 0.886 ± 0.051 r^2^, 0.052 ± 0.009 % mean average error, and 6.884 ± 1.283 kg maximal residual error in FFM estimation. The model further demonstrated its effectiveness in Bland-Altman comparisons with the skinfold thickness-based FFM estimation method, achieving the least mean bias (0.007 kg). The novel TTC model can provide an alternative to full-body BIA measurements, demonstrating an accurate FFM estimation independently of population variables.

## Introduction

Obesity, one of the most crucial problems in healthcare, has risen dramatically in the past few decades [1]. Among the present methods of body composition evaluation, bioelectrical impedance analysis (also known as bioimpedance analysis or BIA), is one of the most widely used, as it provides a quick and efficient way to estimate and control body fat level.

Fat-free mass (FFM), i.e. the difference between fat mass (FM) and total body mass (TBM), is commonly employed by researchers and specialists studying body composition. In general, FFM, as well as FM, can be used as a basis for creating a personal diet plan in order to prevent obesity or muscular dystrophy. Purely anthropometric FFM estimation methods, such as equations derived by Duerenberg [2], Peterson [3], and the Navy Seal formula [4], have limitations, mostly arising from their strong dependency on the traits of the original experimental sample: ethnicity, sex (gender), age, and general health. The gold standard in FFM estimation, dual-energy X-ray absorptiometry (DEXA), has more recently been widely adopted because of high measurement accuracy, accessibility, and short scanning time [5]. DEXA is, however, not suitable for everyday body composition analysis due to equipment size and cost. Moreover, the maximum effective X-ray radiation dose for a single DEXA scan can reach 1.92 μSv, so this method cannot be recommended for frequent use [6].

Thus, BIA can be a way to overcome the challenges of DEXA and anthropometric FFM estimation, since the current production level of the electronics manufacturing industry makes it possible to use it widely. The method utilizes small variations in the human body’s electric conductivity, altered by the impinged frequencies and body composition [7]. Usually, BIA models are formed by training multiple linear regressions, where DEXA results are used as training data (even though DEXA as an FFM estimation method does have its own bias [8]). An example of this approach is the single-cylinder-based BIA model, in which FFM is determined by the value of height^2^/R (known as bioimpedance index, BI), where R denotes the real part of bioimpedance (resistance) between the voltage (sensing) electrodes. The model assumes the human body is an isotropic conductor with a constant cross-sectional area, with the length of the conductor being the height. This makes it necessary to add age and sex variables to the prediction models in order to consider age and sex differences in fat and muscle mass body distribution among the population [9, 10]. However, as the human body has a more complex shape than a single cylinder, the model has severe limitations. For accuracy purposes, the human body can be represented as five cylindrical conductors: four limbs and a torso, excluding the head [8]. With such a five-cylinder model, empirical estimation can be minimized due to direct impedance measurement of body parts. The model's main drawback is the requirement of a multielectrode system, contacting each limb, usually incorporated in a bulky full-size device, which is suitable for the laboratory but not for home use. Although laboratory BIA measuring equipment has multiple advantages such as higher accuracy and inter-observer reproducibility, interest in portable BIA devices, especially wearable ones, has been growing in recent years, mainly due to the growing wearable devices market [11].

In order to analyze upper-body impedance, a wearable wrist-worn impedance meter (WIM) was designed as a modification of AURA Strap, a wrist-wearable Apple Watch accessory (AURA Devices, Inc., USA). The WIM is based on CS1259 (analog front-end with 24-bit ADC designed for body impedance analysis, Chipsea Technologies Shenzhen Corp Ltd, China) and uses a tetra-polar BIA measurement scheme where two electrodes contact with the volar wrist and the other two contact with the free hand. The impedance meter is powered by a CR2025 battery in order to avoid noise from an external power source and maintain autonomous work of the device. Control of the WIM and data transfer are performed via Bluetooth protocol.

In this study, we modified the five-cylinder model into a new model, which includes two upper limbs, for the described bioelectrical impedance meter. With this modification, we expect to keep the advantages of the five-cylinder model, i.e., minimize the empirical estimation of body parts and take additional sex and age variables out of consideration, while employing the hand-to-hand measurement scheme.

Our main aim for the present study was to build a novel non-empirical FFM estimation model based on anthropometric parameters and BIA values measured with the WIM. Since electrode location and measuring frequency affects both BIA accuracy and precision, we also investigated the effect of these factors on the model’s performance. Finally, we compared the non-empirical model’s effectiveness against the population-based model.

## Materials and methods

### The participants

**Fig.1:**
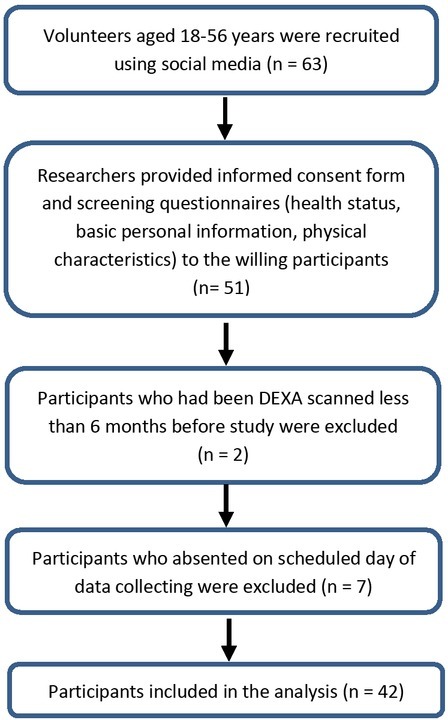
Flow chart of the study participants included in the analysis.

Forty-two healthy adults (21 male, median age: 21.5, age range: 18-56) of Caucasian ethnicity participated in the study (Figure 1). Participants self-reported no recent history of hospitalization, alcohol or diuretic drug consumption. Initial exclusion criteria also included chronic and acute conditions like diabetes mellitus, cancer, renal failure, hepatitis-related diseases, pregnancy, and presence of pacemakers or other electrical implants.

Participants were recruited via an advertisement on social media and were offered financial compensation. They were informed of the experimental purpose, methods, procedures, and safety-related information, and signed the informed consent before participation. The study was conducted in accordance with the Declaration of Helsinki [13].

### Experimental procedure

Before the experiment, all participants were asked to fill out a questionnaire assessing their age, gender, physical training, habitual levels of smoking and alcohol consumption, recent intake of water and caffeine-containing products, normal sleep routine, medication, and menstrual cycle. After filling out the questionnaire, they were asked to take off any metal attachments such as jewelry and piercings and change into cotton medical gowns.

At the first stage of the experiment, a Lunar iDXA (GE Healthcare, USA) whole body scan was taken. After that, TBM and height was assessed with digital scales (resolution: 50 g) and a stadiometer (resolution: 1 mm). Then anthropometric measurements were taken, which included hips, waist, and arm, shoulder and wrist circumferences on both sides. Skinfold thickness was assessed with a caliper at 4 sites (triceps, subscapular, suprailiac and mid-thigh) as described in [3]. Finally, segmental body impedance measurements were performed with InBody 230 (Biospace Co., Ltd., Republic of Korea).

### Bioimpedance measurements

During the experiment, a WIM prototype was placed on the left wrist 2 cm down from the lunate bone. The device has two small 14.2 x 21.9 mm electrodes (300 mm^2^) on each side, which results in a narrow contact area with the skin, and, consequently, a prolonged measurement time in order to obtain a stable bioimpedance value [14]. To minimize this issue, we used a relatively long measurement time (60 sec) and sophisticated signal processing techniques based on two-probe and four-probe measurements.

**Fig.2:**
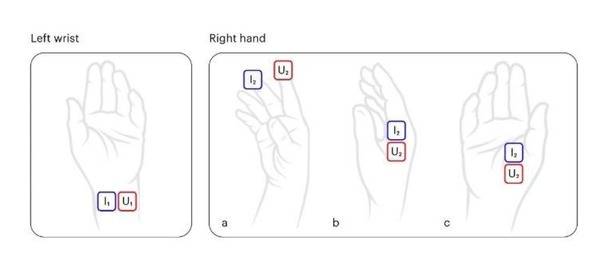
The positions of the WIM electrodes on the left wrist and right hand during bioelectrical impedance measurement.

The upper current and voltage electrodes of the WIM contacted the central zone of the left volar wrist. Three different positions of lower electrodes were used in the study (see Figure 2): A - the index and middle fingers of the right hand; B- the proximal phalanx of the index finger of the right hand; C- the thenar of the right hand.

Hand-to-hand bioimpedance was measured in a standing posture using current frequencies of 10, 50, and 100 kHz for each of the three lower electrode positions. During the measurement, the elbows were flexed to 90 degrees while the hands were at the level of the solar plexus. Every measurement was replicated five times. Researchers monitored the measurement process in real time by computer connection with the WIM via Bluetooth protocol. In order to validate the impedance values obtained with the WIM, all measurements were duplicated with a MAX30001EVSYS (Maxim Integrated, USA). Instrumental error of the WIM is not considered here since it is much less than the errors of the FFM prediction model used.

### Upper body bioelectrical impedance model measurements

**Fig.3:**
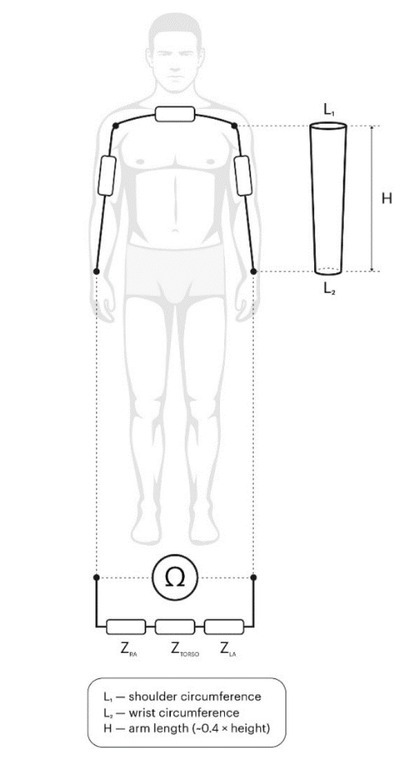
The hand-to-hand bioelectrical impedance scheme of the TTC model.

The truncated cones model is shown in Figure 3, where hand-to-hand bioelectrical impedance consists of two serial impedances of the right and left arm, |Z|_RA_ and |Z|_LA_, and torso impedance |Z|_TORSO_. Although the torso accounts for around ½ of total body mass, its impedance could be ignored due to its low value resulting from high cross-sectional area [8]. The volume of the arms can be represented by the sum of the volumes of two truncated cones, where the shoulder circumference is the length of the base circle and the wrist circumference is the length of the top surface circle. As a result, the new hand-to-hand impedance model for FFM estimation — the two truncated cones (TTC) model — includes the following variables: arm volume, TBM, BI and body mass index (BMI).

### Statistical analysis

Linear regression equations for FFM estimation were built via the least squares method using the Scikit-learn library [15] for Python. In the single cylinder (SC) model, height, age, TBM, sex, and BI were the independent variables, while fat-free body mass obtained by DEXA scan (FFM_DXA_) was the dependent variable. In the new TTC model, arm volume, BMI, and BI were the independent variables, with the same dependent variable. Due to the moderate number of original observations (N = 42), the bootstrap resampling method with 1,000,000 samples was used to derive regression coefficients (mean training sample size n_train_ = 26.75, mean testing sample n_test_ = 15.25). Coefficient of determination (r^2^), mean absolute percentage error (MAPE), and maximal residual error were the metrics of choice to evaluate both models’ performance.

In order to investigate the agreement between FFM estimates provided by the SC and TTC models with the DEXA reference, a Bland–Altman plot was created. Mean biases with 95 % confidence intervals were calculated as the main measures of agreement between the results, and ± 1.96 standard deviations were set as the limits of agreement (LOA).

### Informed consent

Informed consent was obtained from all individuals included in this study.

### Ethical approval

The research related to human experimentation complied with all relevant national regulations and institutional policies, and was in accordance with the tenets of the Helsinki Declaration.

## Results

### Anthropometry and BIA

The results of the anthropometric measurements and calculations, DEXA scan, and WIM BIA measurements can be seen in Table 1. The mean (± STD) age of male and female participants was 24.95 ± 8.0 years and 29.1 ± 11.65 years, respectively. The physical condition of the participants was rather broad, from athletic to moderate obesity: the BMI and body fat percentages of the males were (23.69 ± 2.29) kg/m^2^ and (20.35 ± 7.55) % respectively, while the BMI and body fat percentages of the females were (23.51 ± 5.13) kg/m^2^ and (33.18 ± 5.9) % respectively.

**Table 1:** Anthropometric, body composition and bioelectrical impedance data.

	Mean ± STD	Range
Age, years	27.02 ± 10.2	18 - 56
TBM, kg	69.56 ± 11.29	45.83 - 100.95
Height, cm	171.89 ± 8.86	156.0 - 196.0
BMI, kg/m^2^	23.6 ± 3.97	18.17 - 38.87

Total arms	6109.06 ± 1505.04	3480.6 - 10970.2
volume, cm^3^		

FFM_DEXA_, kg	50.99 ± 10.94	34.51 - 86.59
FM_DEXA_, %	26.77 ± 9.34	8.32 - 48.39

Re(Z)_10_a_, Ohm	633.13 ± 136.83	366.43 - 1001.8
Re(Z)_10_b_, Ohm	559.43 ± 169.33	88.28 - 866.89
Re(Z)_10_c_, Ohm	627.71 ± 133.16	398.16 - 964.93
Re(Z)_50_a_, Ohm	592.86 ± 134.54	379.79 - 914.78
Re(Z)_50_b_, Ohm	551.97 ± 160.0	126.64 - 889.12
Re(Z)_50_c_, Ohm	562.21 ± 138.21	304.83 - 831.84
Re(Z)_100_a_, Ohm	571.13 ± 133.97	352.69 - 881.58
Re(Z)_100_b_, Ohm	553.53 ± 141.96	312.6 - 890.5
Re(Z)_100_c_, Ohm	527.9 ± 134.54	204.77 - 799.5

* Re(Z)**_x_y_**= reactance of bioelectrical impedance measured at frequency of **x** kHz and with right hand position **y** shown in Fig. 2

### Upper body bioimpedance approximation with arm volume

**Fig.4:**
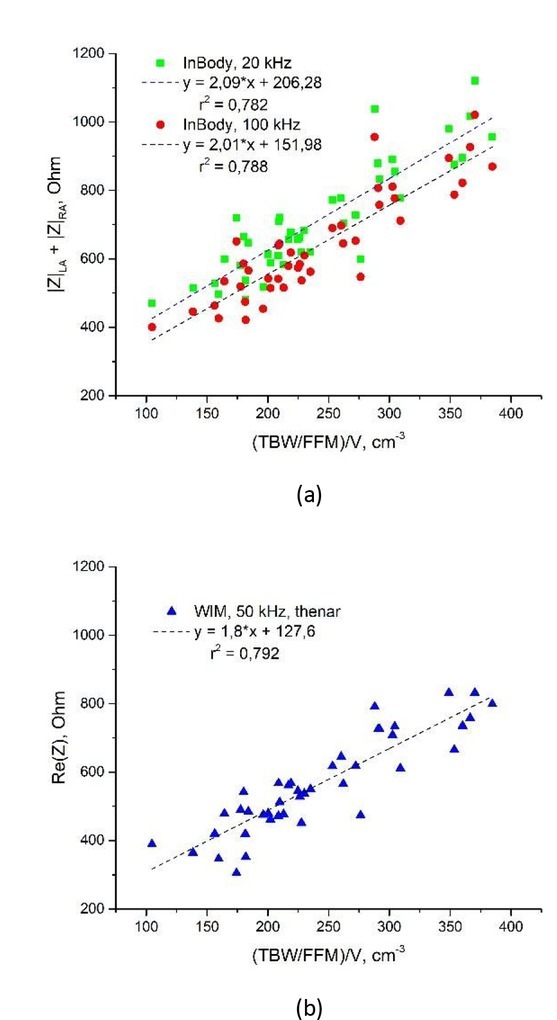
Arm volume multiplied by TBM/FFM ratio approximates bioimpedance: (a) Approximation of InBody 230 arm impedance. (b) Approximation of real part of bioimpedance measured with WIM.

Arm volume multiplied by TBM/FFM ratio can be used to approximate the sum of the arms’ impedance determined by the five-cylinder human body model used in InBody 230 [16] (Figure 4a). The coefficient of determination r^2^ was 0.782 and 0.788 for the sum of left and right arm impedance (|Z|_LA_ + |Z|_RA_) measured at 20 kHz and 100 kHz, respectively. Thus, changes in bioimpedance imply changes in geometry of measured limbs. The higher value of r^2^ (0.792) in the case of WIM Re(Z)_50_c_ approximation (measurements taken at 50 kHz, right hand electrode position c), can be interpreted as arm impedance making the main contribution to the value of the real part of bioimpedance. These results provided additional grounding to the TTC model, as total arm volume was used as an independent variable in FFM estimation.

### FFM estimation regression and effect of measuring frequency and electrode position

Two linear regressions were built in this study: one for the SC model and one for the TTC model. For both models, regressions built on 50 kHz electrode position c data demonstrated the lowest MAPE and maximal absolute error as well as the highest coefficient of determination r^2^ (Table 2).

**Table 2:** Linear regression metrics for FFM estimation: two models (SC and TTC) and nine experimental conditions

Frequency, kHz	Right hand electrode position	SС model			TTC model		
		r2	MAPE	Max. err.	r2	MAPE	Max. err.
10	a	0.656 ± 0.209	0.076 ± 0.016	15.732 ± 5.076	0.679 ± 0.211	0.075 ± 0.017	14.404 ± 4.756
10	b	0.605 ± 0.487	0.085 ± 0.023	15.088 ± 6.327	0.514 ± 0.37	0.096 ± 0.022	17.862 ± 6.684
10	c	0.777 ± 0.114	0.065 ± 0.012	12.463 ± 4.224	0.795 ± 0.112	0.064 ± 0.013	11.236 ± 3.888
50	a	0.763 ± 0.119	0.064 ± 0.014	12.493 ± 3.357	0.774 ± 0.125	0.062 ± 0.014	12.16 ± 3.456
50	b	0.479 ± 0.309	0.111 ± 0.309	27.447 ± 24.079	0.422 ± 0.338	0.119 ± 0.338	30.852 ± 25.537
50	c	0.876 ± 0.054	0.051 ± 0.009	8.623 ± 2.596	0.886 ± 0.051	0.052 ± 0.009	6.884 ± 1.283
100	a	0.752 ± 0.131	0.065 ± 0.014	12.883 ± 3.534	0.762 ± 0.14	0.064 ± 0.015	12.595 ± 3.507
100	b	0.698 ± 0.143	0.078 ± 0.015	13.653 ± 3.794	0.761 ± 0.133	0.07 ± 0.014	11.829 ± 2.888
100	c	0.482 ± 0.423	0.086 ± 0.02	20.076 ± 9.376	0.435 ± 0.461	0.094 ± 0.021	20.44 ± 10.855

Thus, the final linear regression equations for the single cylinder (FFM_SC_) and TTC (FFM_TTC_) models were as follows:

*FFM_SC_ = 2.835 × sex – 0.102 × age + 0.261 × TBM + 0.425 × BI + 10.105*

*FFM_TTC_ = 182.858 × (arm volume) – 0.796 × BMI + 0.464 × TBM + 0.435 × BI + 11.729*

**Fig.5:**
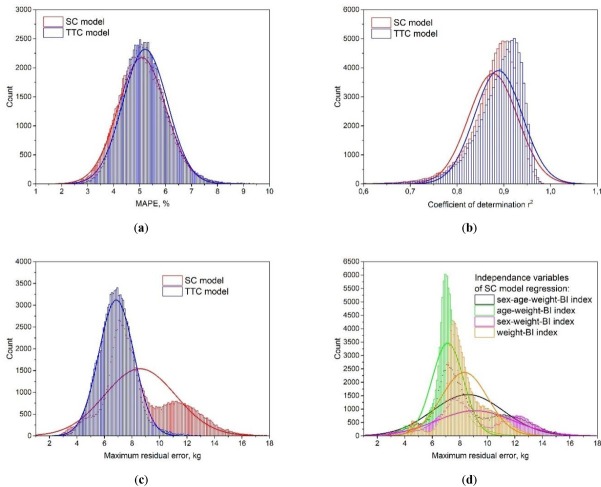
(a) MAPE; (b) Coefficient of determination r^2^; (c) Maximal residual error. Distribution shapes were obtained during 100,000-sample bootstrap procedure. Bold lines show normal approximation curves; (d) The effect of sex and age variables on maximal residual error distribution for the single cylinder model.

As can be seen in Figure 5a,b, the TTC model showed a higher r^2^ and an almost identical MAPE distribution among the bootstrap samples. However, the two models’ distribution of maximal residual error demonstrated drastically different shapes (see Figure 5c). The TTC model error followed close to normal distribution, while the SC model error had a more complex multimodal distribution. The appearance of several peaks was likely caused by different multilinear regression independent variables or the interaction between them. Figure 5d shows the effect of removal of sex, age, and both variables on maximal residual error distribution.

The absence of the sex variable resulted in the disappearance of the higher error peak, which can be a sign of the model performing accurately for only one value of this variable. Interestingly, the small peak of lower residual error was apparently not influenced by either sex or age variables and can probably be interpreted as the result of more complex or higher-order variable interaction.

### Bland-Altman plots

**Fig.6:**
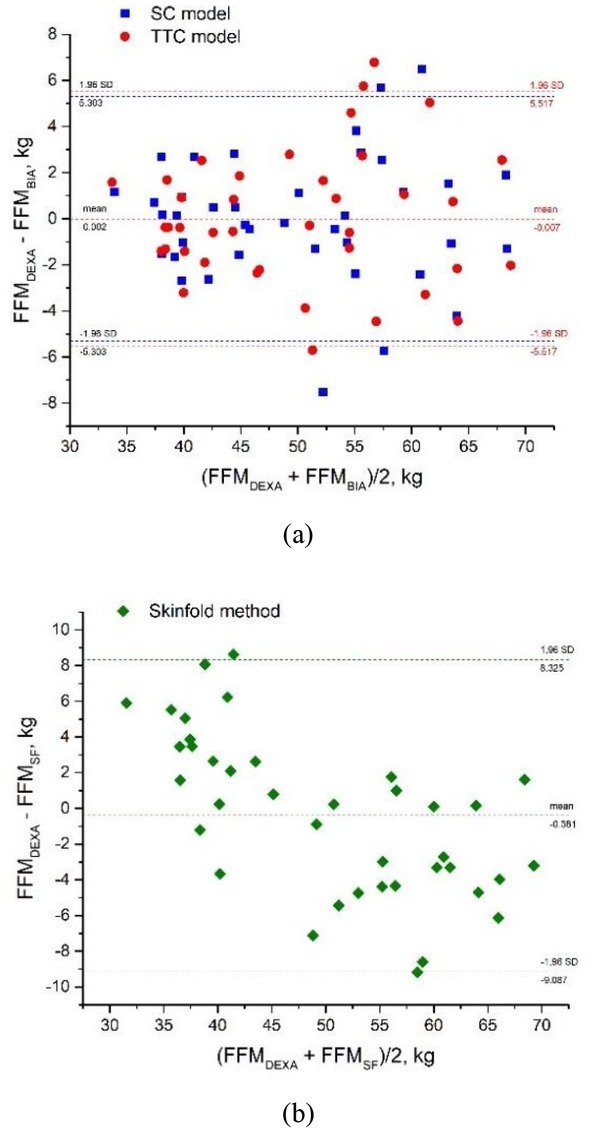
Bland-Altman plots for WIM FFM estimate validation: (a) Single cylinder (SC) model: confidence intervals for the mean bias: (-0.874, 0.879) kg, for upper LOA: (4.380, 6.937) kg, for lower LOA: (-6.932, -4.375) kg; TTC model: for mean bias: (-0.920, 0.905) kg, for upper LOA: (4.547, 7.208) kg, for lower LOA: (-7.223, -4.562) kg. (b) Skinfold thickness calculations: confidence intervals for the mean bias: (-1.792, 0.955) kg, for upper LOA: (6.653, 10.651) kg, for lower LOA: (-11.488, -7.4918) kg.

The Bland-Altman plots in Figure 6a demonstrate the agreement between the SC and TTC model FFM estimation and the reference method (DEXA). As can be seen, the mean bias between estimates was only -0.007 kg, while the limits of agreement were (-5.517; 5.517) kg, which indicated good accordance. A Bland-Altman plot was also built for FFM estimates provided by the equation based on skinfold thickness measurements [3] (Figure 6b). Although skinfold-based method calculations reached fine agreement with reference values (-0.381 kg mean bias), its LOA (-9.087; 8.325) kg were much broader.

## Discussion

In this study, we developed the novel TTC model of FFM estimation based on hand-to-hand BIA measurements made by a wrist-worn impedance meter. We also investigated the effects of measuring frequency and electrode positions on model performance and demonstrated the agreement between the model and reference DEXA estimates of fat-free mass.

We derived both empirical and non-empirical regression equations based on the single cylinder and TTC models. One of the main advantages of the TTC model turned out to be the uniformity of distribution of maximal residual error. The population-based single cylinder model error followed a multimodal distribution with several peaks resulting from the influence of the sex variable. In practical use, this can lead to a significant FFM prediction error. The TTC model, on the other hand, did not have this disadvantage.

Bioimpedance values (Table 2) measured using different placements of the WIM electrodes and at different frequencies varies. The highest value was measured through the fingers (Figure 2a) for all frequencies since they represent the longest body pathway among the ones tested. Measurement frequency also affects the bioimpedance value: a 10 kHz current resulted in the highest resistance for all electrode placements.

The effect of measuring frequency and electrode placements on the performance of FFM models was also analyzed. The bioimpedance measured through the proximal phalanx of the index finger of the right hand (Figure 2b) resulted in the worst performance with the lowest r^2^ and the highest MAPE. Although measurements taken through the index and middle fingers showed better performance, it was still low compared with measurements taken through the thenar. This could be related to the high influence of a non-relevance additional impedance of the whole hand including contacting fingers, whose impedances do not strongly depend on a person’s body composition.

It was found that 50 kHz measurements on the right hand thenar (Figure 2a) resulted in the lowest MAPE and maximal residual error and the highest r^2^ for both models. It is possible that the higher amount of muscle tissue (e.g., flexor pollicis brexis, adductor pollicis brevis) under the thenar electrode is associated with more stable values of measured bioelectrical impedance, probably due to noise reduction and lesser effect of skin conductance.

Comparison of the SC and TTC models’ performance with the results obtained by DEXA scan demonstrated the high accuracy of both models, with mean bias between the methods being not significantly different from zero. These results demonstrating the new TTC (as well as SC) model for the WIM suggest that it can provide an accurate and effective method to assess body fat-free mass.

There are, however, limitations to the uses of the new models, the main one being related to the physical principles behind bioimpedance analysis. As only single-frequency BIA (50 kHz) measured impedance was utilized in building the models, their ability to distinguish between extracellular and intracellular water is hindered. As a result, FFM estimation could be easily affected by electrolyte imbalances or body hydration status [17]. As a typical empirical BIA model, our single cylinder model depends on the sex, age, and ethnicity of the experimental sample, and can possibly perform worse for the rest of the population. The TTC model is non-empirical but has a practical limitation due to the requirement of specific measurements, such as wrist and shoulder circumferences.

In the future, the TTC model can be enhanced by employing bioimpedance spectroscopy (also known as multifrequency BIA), since the use of several frequencies makes it possible to differentiate total body water into extracellular and intracellular spaces and avoid the effect of body hydration status on FFM prediction [18]. Moreover, since the WIM prototype is able to assess phase angle, as well as both the real (resistance) and imaginary (reactance) part of bioimpedance, this data can be used to reveal water distribution between the extra- and intracellular spaces [19]. This will further improve the performance of the TTC model. Last but not least, the geometrical shape of the arms can be more closely approximated: instead of a truncated cone, each arm can be represented as a more realistic complex geometrical object consisting of several truncated cones.
